# Forceps-Assisted Delivery Could Reduce the Iatrogenic Fistula Crisis: A Clinical Commentary

**DOI:** 10.1007/s00192-026-06640-5

**Published:** 2026-05-22

**Authors:** Hannah G. Krause, Magdalena N. Halt, Catherine A. Matthews, Judith T. W. Goh

**Affiliations:** 1 University of Queensland School of Medicine, Brisbane, QLD Australia; 2 Gold Coast University Hospital, Gold Coast, QLD Australia; 3 Greenslopes Private Hospital, Suite 209 Ramsay Specialist Centre, Brisbane, QLD Australia; 4 Royal Adelaide Hospital, Adelaide, SA Australia; 5 Wake Forest University School of Medicine, Winston Salem, NC USA; 6 Griffith University School of Medicine, Gold Coast, QLD Australia

**Keywords:** Forceps, Maternal morbidity, Obstructed labour, Second-stage arrest, Ureterovaginal fistula, Vesicovaginal fistula

## Abstract

In an era where the aetiology of childbirth-related pelvic floor fistula is rapidly shifting from ischaemic obstetric fistula to iatrogenic childbirth-related fistula related to complications of caesarean section, strategies must be implemented to minimise the ongoing burden of fistula-related pathologies and surgical morbidity and mortality. Despite growing access to caesarean section in low- and middle-income countries (LMICs), this mode of delivery has introduced new maternal complications such as ureterovaginal and vesico-uterine fistulae that often require abdominal repair. The reintroduction of the safe and skilled use of forceps for delivery in cases of second-stage arrest with the foetal head well below the level of the ischial spines, in particular, cases of intra-uterine foetal demise and exhausted maternal effort, may assist in reducing this trend. In addition to improving surgical training in caesarean section skills, the reintroduction of comprehensive training for vaginal instrumental deliveries including forceps and further emphasis on providing effective obstetric management, may help to improve maternal health care and is urgently needed.

## Introduction

The face of obstetric fistula is changing. Historically, these injuries have arisen from prolonged obstructed labour, resulting in ischaemia and necrosis of the soft tissues in the pelvis [1]. Over the past 15 years, however, the proportions of iatrogenic genitourinary fistulae in sub-Saharan Africa, primarily following caesarean section, have increased from 9.6% of total genitourinary fistulas in 2010 to 43.2% in 2020 and have been attributed to limited training of house officers performing abdominal delivery [2]. Significantly improved access to obstetric services in LMICs has unfortunately not reduced genitourinary fistulae associated with childbirth, but has rather changed their aetiology from ischaemic to iatrogenic [3].

The proposed solution to reversing this iatrogenic trend has focused on improving caesarean section competency. Presented as the prizewinning abstract at the International Urogynecology Association meeting in 2025, Byamukana et al. [4] detailed results of the Essential Training in Operative Obstetrics (ETTO) intervention in Uganda, a formalized programme of caesarean section simulation to improve competency amongst junior officers. The term “operative obstetrics” in this intervention applied exclusively to abdominal delivery. This study is representative of many LMICs where vaginal instrumental deliveries, including obstetric forceps and vacuum extraction, have largely been abandoned [5]. Therefore, for women in the second stage of labour with an indication for assisted delivery, the only option is emergency caesarean section, with its associated risks and complications.

Operative vaginal delivery, specifically with forceps, is a potentially safer option for uterine evacuation in the event of second-stage arrest, with the foetal head well below the level of the ischial spines and suitable for vaginal delivery, particularly in cases of intra-uterine foetal demise or maternal exhaustion. Obstetric forceps, invented in the sixteenth century and progressively optimised, continue to save lives [6]. This commentary is aimed at contextualising the indications, risks and benefits of forceps delivery in non-tertiary LMIC settings and presents the rationale for restoring this tool as a necessary component of “operative obstetrics” for the primary intention of reducing the risks of iatrogenic urogenital fistulae.

## Terminology

With the recent change in aetiology of obstetric fistulae, new terminology has been introduced to clarify contributing factors to presentation [1]. The term obstetric fistula specifically refers to ischaemic injury from obstructed labour. Iatrogenic childbirth-related fistula (ICBRF) describes fistulae resulting from injuries to the urinary or anorectal tracts during operative delivery, including caesarean section, caesarean hysterectomy or operative vaginal delivery including episiotomy [1]. Recent publications [7, 8] have demonstrated the dramatic increase in iatrogenic urinary-tract fistulae secondary to caesarean section, highlighting serious concerns regarding the quality of obstetric operative care. Although ICBRFS occur worldwide at all levels of resource availability, they are more prevalent in LMICs, where there is a lack of skilled and experienced surgeons performing these procedures.

Mixed obstetric and iatrogenic fistula (MOIF) refers to fistula related to operative delivery for prolonged obstructed labour. This is where tissue integrity has already been compromised by the obstructed labour before the operative delivery. There are currently no published data on the incidence of this fistula aetiology. Determining the cause of a fistula following caesarean section during labour is often presumptive, taking into consideration the duration of labour, the degree of obstruction prior to delivery, and the status of tissue integrity at the time of surgery. As obstetric fistula only becomes evident once tissue sloughs away following the necrotic insult, it may be diagnosed following the caesarean section. Differentiating obstetric fistula or MOIF from ICBRF [1] may therefore be difficult.

## Rise of Caesarean Sections in Low- to Middle-Income Countries

Obstructed labour, particularly second-stage arrest, presents a dilemma to both patient and accoucheur. Left untreated, it contributes to ischaemic obstetric fistula, which affects both the genitourinary and the anorectal tracts [1]. Prior to the development of instrumental deliveries and caesarean section, obstetric fistula was common throughout all economies, with the first women’s hospital dedicated to surgical fistula repair opened in New York City in 1855 [9]. With access to operative obstetrics, both vaginally and abdominally, obstetric fistula has become very infrequent in high-income countries. Unfortunately, these injuries persist in LMICs, where resources dedicated to effective obstetric management continue to be limited [10, 11].

Improving access to obstetric services has been a focus for many LMIC countries over the past 2 decades. For example, Rwanda and Uganda spearheaded campaigns focused on improving maternal morbidity and mortality and intentionally increased access to health care facilities for labour management. Correspondingly, caesarean section rates in Rwanda have risen from 3% in 2005 to 15% in 2019 according to the Rwanda Demographic and Health Survey [12], whereas Tanzania increased from 8 to 13% between 2012 and 2022 [13] and Ugandan rates are now comparable at 13.4% (for 2019/2020) [14].

## Unintended Consequences of Second-Stage Caesarean Section

Maternal complications following caesarean section are three-fold higher than after vaginal delivery [15]. Second-stage arrest presents a formidable challenge, as all methods of delivery can be associated with morbidity. Although the overall safety of caesarean section has markedly improved over recent decades, substantially higher risks remain for caesarean section performed for second-stage arrest and substantial experience and expertise are required to minimize morbidity [16]. Not only do the risks of caesarean section surgery pertain to the immediate procedure [17–19] but there are also implications for subsequent pregnancies and deliveries.

Maternal risks of second-stage caesarean section include major haemorrhage, longer hospital stay, greater risk of bladder and ureteral trauma, and extension tears of the uterine angle leading to broad ligament haematoma [16, 20]. These are even more likely to occur when tissue necrosis is in progress. Infectious morbidity is more prevalent in second-stage caesarean section than in instrumental delivery [21]. Future pregnancies may be complicated by abnormal placentation, antepartum bleeding, uterine rupture and damage to surrounding organs, particularly the bladder [22]. As such, the World Health Organization recommends avoidance of this mode of delivery without clear medical indications [20, 23]. Fawcus et al. [24] detailed the increasing incidence of maternal deaths secondary to bleeding associated with caesarean delivery in South Africa over the last 20 years. The case fatality rate per 100,000 caesarean deliveries in the triennium 2002–2004 was 20.9, increasing to 33.6 in the 2011–2013 triennium. The authors describe maternal deaths from bleeding with caesarean delivery as a national emergency and request the urgent activities needed to reduce such deaths, including prevention, early recognition, immediate resuscitation and treatment to stop the bleeding, emergency transport and leadership.

Although the prevalence of caesarean section has risen dramatically in LMICs, this has not translated into lower rates of obstetric fistulas or improved prevalence of live births [25]. For example, in 1996, Goh and Krause retrospectively evaluated 100 women with obstetric fistulas and reported a 13% caesarean section and a corresponding 5% live birth rate [26]. In 2015, they conducted a similar audit of 100 obstetric fistula repairs in central Africa and reported a rise in the caesarean section rate to 54%, yet the live birth rate remained at 6%. Most recently (2025), the last 100 obstetric fistula repairs performed by the author (JG), also in central Africa, recorded a caesarean section rate of 65% and a live birth rate of 7%.

In addition to no measurable improvement in live birth rates for women with second-stage arrest who develop obstetric fistula, cesarean delivery who develop obstetric fistula, caesarean delivery has further unintentionally introduced the new problem of ICBRF [2, 27, 28]. Lack of training and inexperienced surgical teams are resulting in rising rates of ICBRF, which are typically high fistulas to the uterus or cervix or involve the ureters and more commonly require abdominal repair [3, 17, 29, 30]. A recent retrospective review of obstetric fistulas presenting over 10 years in Kigali, Rwanda demonstrated a 2.1% increase per year of iatrogenic fistula associated with additional surgical complexity and complications [3].

## Potential Benefits of Obstetric Forceps Delivery for Second-Stage Arrest, Especially in Cases of Intra-Uterine Foetal Demise

There is a global trend where operative vaginal delivery has gradually fallen out of favour among obstetricians in the past three decades and now makes up about only 1.1% of vaginal deliveries [31]. However, in Australia and New Zealand, instrumental vaginal births comprise around 11.6% of all deliveries, with emergency caesarean sections amounting to approximately 13% (total caesarean section rate 39%) [32]. Reliable and contemporary rates of instrumental deliveries across LMICs, particularly Sub-Saharan Africa, are difficult to source, but have been quoted to be as low as 1% to a high of 12% and are site dependent [33]. Recent analysis of 17 studies reporting on instrument-assisted deliveries across Ethiopia, Nigeria, Mali, Senegal, and Cameroon reported rates of just under 8% of births, and emergency caesarean section accounting for 5% of deliveries [11, 34].

Although birth attendants have used forceps for centuries and vacuum assistance for decades, recent delivery trends have demonstrated a dramatic decline in forceps use with a corresponding rise in caesarean delivery rates [35, 36]. Some speculate that this downward trend is due to fear of litigation related to common complications of forceps delivery, including increased risk of perineal laceration and injury to the newborn. Others attribute the decreased use of forceps to a lack of experienced personnel in forceps delivery who can conduct adequate training [37] and fear regarding injury to mother and foetus. In the USA, a sharp decline in forceps training has occurred as a significant proportion of senior obstetrics and gynaecology residents no longer feel comfortable performing forceps deliveries [38], with many teaching hospitals performing this type of delivery rarely or not at all [39].

Education is paramount to any operative intervention and equally applies to forceps-assisted delivery. The safe use of forceps requires appropriate patient selection and proper application to reduce risk of injury [40]. In cases of intra-uterine foetal demise, neonatal risks of operative vaginal delivery obviously no longer apply, although vacuum extraction may not be suitable if foetal scalp tissue necrosis is present. The scalp suction required during vacuum extraction may result in the tissue separating from the skull with loss of suction.

## Acknowledging the Risk of Forceps-Assisted Delivery

The premise of this commentary is to advocate for the re-introduction of operative vaginal deliveries as an alternative to caesarean section in clinically appropriate circumstances; however, only in the setting of effective training programmes whereby the clinicians performing patient assessment and instrumental vaginal deliveries are skilled in these techniques. Operative vaginal deliveries must not be performed in the absence of competency, otherwise serious risks of foetal and maternal morbidity and mortality may ensue.

The maternal risks of operative vaginal delivery include cervical laceration, pelvic-floor injury, and more extensive perineal lacerations, especially in the absence of adequate training [41]. In a recent systematic review and meta-analysis of 3754 patients from Ethiopia, the maternal complication rate from instrumental delivery (including perineal and cervical tears, obstetric anal sphincter injuries, traumatic post-partum haemorrhage and uterine rupture) was 21% [42]. Comparable complication data for Canada showed 13.2% maternal complications [43], which likely reflects differences in training. It is plausible that this high rate of observed complications in LMICs prompted the abandonment of forceps. Responsible forceps reintroduction will require advanced education, including high-fidelity simulation.

Ongoing studies documenting a high risk of levator ani muscle trauma following forceps deliveries [44], and the association of levator ani muscle trauma with pelvic organ prolapse [45], have more recently been major factors in the reduction of utilization of forceps in global obstetric care. Since the Oxford Family Planning Study in 1997 [46], it has been clearly identified that increasing vaginal parity is the strongest risk factor for pelvic organ prolapse in women younger than 60 years. However, it is the first vaginal delivery that results in the highest risk of levator ani muscle trauma [47]. Although it is uncommon for complete levator ani muscle defects to occur following caesarean section, even in women who have laboured [48], this has been reported [49], including in women with obstetric fistulae [25]. Krause et al. [25] report an overall rate of complete levator ani muscle defects in women with stages 3 and 4 (POPQ) pelvic organ prolapse of 21.1%. In the same study, women with obstetric fistulae had complete levator ani muscle defects in 28.6%, with 71.4% of the primiparous women with obstetric fistula having had a caesarean section. None of the women with obstetric fistula had pelvic organ prolapse greater than stage 1 (POPQ) [50]. Reasons for the development of pelvic organ prolapse are multifactorial, with use of forceps increasing the risk of levator ani muscle trauma being just one contributing factor. Therefore, where labour is already very prolonged, the avoidance of forceps delivery may not necessarily result in protection of the pelvic-floor muscles, and complications from emergency caesarean section in the second stage may be more damaging.

As rates of human immunodeficiency virus infection worldwide increased alarmingly in the 1990 s, and without effective anti-retroviral therapies available, the use of instrumental deliveries, in particular vacuum extraction, was restricted owing to the risk of the vertical spread of human immunodeficiency virus to the foetus during delivery. In the absence of intervention, a systematic review of 13 studies places the risk of transmission from a mother with human immunodeficiency virus to the child during pregnancy, labour or delivery at between 15 and 30% [51]. With effective use of anti-retroviral therapies and post-exposure prophylaxis, rates of vertical transmission have dramatically reduced for all modes of delivery. International guidelines document management advice for women with human immunodeficiency virus infection during pregnancy and labour, stating that where human immunodeficiency virus viral load is undetectable, vaginal delivery does not increase the risk of human immunodeficiency virus transmission to the infant [52]. In cases of intra-uterine foetal demise requiring assistance with delivery in the second stage of labour, human immunodeficiency virus status does not impact the use of forceps.

Although operative vaginal delivery involves a risk of maternal and neonatal complications, many women elect for operative vaginal delivery given the longer recovery and downstream health effects associated with caesarean section [22].

## Call for Action: Re-Introduction of a Safe Trial of Forceps for Second-Stage Arrest, Especially for Intra-Uterine Foetal Demise

The rising national trends in caesarean sections in LMICs and programmes such as the ETTO intervention in Uganda [4] are emblematic of the disappearing place of forceps from the toolbox of obstetricians and birth attendants. The consequences of this disturbing trend are evident in rising maternal morbidity, especially with regard to iatrogenic urogenital fistulae. Re-introduction of forceps-assisted delivery for second-stage arrest, where the cervix is fully dilated and the foetal head well below the level of the ischial spines, particularly in cases of intra-uterine foetal demise and maternal exhaustion, could reverse this trend. However, it must be done correctly. Not only must clinicians be trained in the technical aspects of forceps-assisted delivery but also in the clinical assessment and indications for proceeding with operative vaginal delivery. Without the appropriate training, forceps could induce similar harm to caesarean section. For example, data from Ethiopia published in 2023 reported a 28.4% rate of maternal complications [53], which is similarly concerning. The markedly increased rates of forceps complications in LMICs have been attributed to limited education and training, local expertise, fear of neonatal complications, equipment shortages and concern about the transmission of infective diseases [11, 33]. These data clearly highlight the urgent need for education and training in the active management of labour, which includes the indications and application of instrumental deliveries.

Simulation training with simple models has proven effective at decreasing severe perineal tears [54]. Organisations such as the Royal College of Obstetricians and Gynaecologists, and the Federation of International Obstetricians and Gynecologists, could develop effective interventions, including a forceps curriculum and simulation programme designed to optimise patient selection and the safe application of forceps. It is time for the forceps pendulum to swing in the other direction. Patients should be offered appropriate management options, with shared decision-making prioritised. Particularly with regard to second-stage arrest with intra-uterine foetal demise, where the foetal head is well below the level of the ischial spines and suitable for vaginal delivery, skilled application of forceps for delivery is most appropriate. The rising trend of iatrogenic urogenital fistulae could be improved or reversed with appropriate training, resulting in the safe reintroduction of forceps.

## Data Availability

No new data were created or analyzed in this study. Data sharing is not applicable to this article.
